# Intraepithelial Macrophage Expressing CD163 Is a Histopathological Clue to Evaluate the Malignant Potency of Oral Lichenoid Condition: A Case Report and Immunohistochemical Investigation

**DOI:** 10.3390/diagnostics10090624

**Published:** 2020-08-23

**Authors:** Manabu Shigeoka, Yu-ichiro Koma, Maki Kanzawa, Masaya Akashi, Hiroshi Yokozaki

**Affiliations:** 1 Division of Pathology, Department of Pathology, Kobe University Graduate School of Medicine, 7-5-1 Kusunoki-cho, Chuo-ku, Kobe 650-0017, Japan; koma069601119@gmail.com (Y.-i.K.); hyoko@med.kobe-u.ac.jp (H.Y.); 2 Division of Diagnostic Pathology, Department of Pathology, Kobe University Graduate School of Medicine, 7-5-2 Kusunoki-cho, Chuo-ku, Kobe 650-0017, Japan; makiron.wtks@gmail.com; 3 Division of Oral and Maxillofacial Surgery, Department of Surgery Related, Kobe University Graduate School of Medicine, 7-5-2 Kusunoki-cho, Chuo-ku, Kobe 650-0017, Japan; akashim@med.kobe-u.ac.jp

**Keywords:** macrophage, CD163, malignant potency, oral lichenoid condition, biopsy

## Abstract

Oral lichenoid conditions (OLC), including oral lichen planus (OLP), oral lichenoid lesions and oral lichenoid dysplasia, differ in pathogenesis and biological malignancy. However, distinguishing them based on clinical or histological features is difficult. It is well known that CD163^+^ macrophages are associated with oral cancer aggressiveness. We recently demonstrated that CD163^+^ macrophages of noncancerous lesions infiltrate the stroma, not the intraepithelial area. In this report, we describe a case of OLC that was not detected as malignant by the first local biopsy. Furthermore, we evaluated the malignant potency of OLC by retrospectively comparing the histological findings between local biopsy and resected specimens focusing on CD163^+^ macrophages. A 72-year-old man with a white lesion in the unilateral buccal mucosa was diagnosed with OLP through the biopsy although invasive cancer was detected two years later. Intraepithelial CD163^+^ macrophages were found not only on the resected specimen but also biopsy. This is the first report to demonstrate that intraepithelial CD163^+^ macrophages may be noteworthy indicators to identify the malignant potency of OLC.

## 1. Introduction

Oral lichenoid conditions (OLC) include oral lichen planus (OLP), oral lichenoid lesions (OLL) and oral lichenoid dysplasia (OLD), and their clinical and histological features overlap [1]. Oral squamous cell carcinoma (OSCC) passes through oral malignant potency disorders (OPMDs) [2]. OLP is one of the OPMDs. However, OLL and OLD are not described in detail by the World Health Organization classification of tumors [2].

OLP, OLL, and OLD differ in etiology, pathogenesis and biological behavior, whereas all of them have close clinical and histopathological resemblances. Therefore, the malignant potency of these lesions is a greatly controversial issue [3]. In fact, the rate of malignant transformation of OLP patients varies considerably in the literature [4]. OLP is a chronic inflammatory mucosal disease characterized by various symptoms. OLL can be caused by dental materials, an array of drugs, graft versus, host diseases or unclassified causative agents [1]. Conversely, OLD is a lesion mainly based on epithelial atypia and should be distinguished from OLP, which is an inflammatory lesion [5]. The accurate distinctions of OLCs have not yet been established. Thus, an efficient method to evaluate the malignant potency of OLCs is desired.

Ki-67 and p53 are biological malignant factors of oral atypical epithelium and OSCC [6]. In normal oral epithelium, Ki-67^+^ cells and p53^+^ cells are sporadically confirmed in the second basal layer. On the other hand, in malignant epithelium, several Ki-67^+^ cells are distributed in the basal and/or more superficial layers [7]. A variation in expression pattern of p53^+^ cells is observed. However, the usefulness of these markers in OLCs has not been completely established because altered expression is observed in the reactive lesion [6].

Macrophages are known to be involved in the aggressiveness of various cancers, and it was revealed that CD163^+^ macrophages predict poor prognosis in patients with OSCC [8]. Moreover, the significance of macrophages expressing CD163 in tongue leukoplakia, one of the most common OPMDs, was recently reported [9]. We also demonstrated that CD163^+^ macrophage infiltration occurs in the oral epithelium depending on the presence of stromal invasion [9]. However, few studies have evaluated malignant potency of OLC focusing on macrophages.

This study reports OLC in a Japanese man diagnosed with a compatible inflammatory change, diagnosed as OLP based on the first biopsy but diagnosed with OSCC following a total resection two years later. Here, we conducted a retrospective immunohistochemical investigation on CD163 using both biopsy and resected samples to evaluate the malignant potency of this OLC.

## 2. Clinical Findings

**Figure 1:**
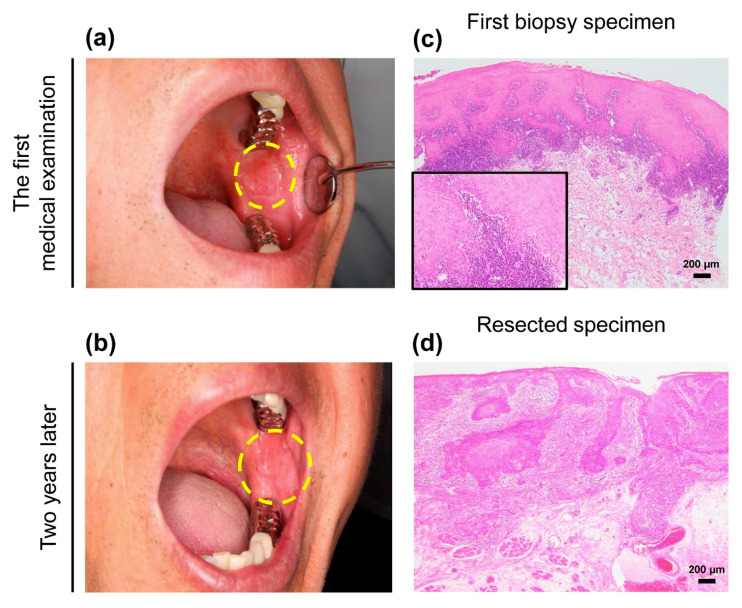
Clinical and morphological features of the patient.(**a**) A unilateral white lesion with a size of approximately 10 × 10 mm in maximum diameter was found in the left buccal mucosa at the first medical examination. The lesion was clinically diagnosed as oral lichen planus (OLP). Dotted line indicates extent of lesion. (**b**) Enlarged white lesion, measuring approximately 28 × 25 mm, with erythematous and induration on the same region of the buccal mucosa appeared two years after the first biopsy. Dotted line indicates the extent of the lesion. (**c**) Morphological evaluation based on the first biopsy showed evidence of lichenoid infiltration and aggression to the basal membrane. The degree of epithelial dysplasia with acanthosis was mild. The histological diagnosis was “compatible inflammatory changes as OLP” (hematoxylin and eosin (H&E)) (**d**) The consecutive resected specimens confirmed invasive cancer with a lymphocytic band around the tumor (H&E). The histological diagnosis was “pT2N0M0 squamous cell carcinoma, well-differentiated (28 × 25 × 2.2 mm)”. Scale bars in (**c**) and (**d**) = 200 µm. Inset in c shows high power view of image.

A 72-year-old man complained to his dentist of whitish painful changes in the left buccal mucosa and was referred to our hospital. A white lesion was found in his left buccal mucosa (Figure 1a). He was clinically diagnosed with OLP and the first local biopsy was performed. The histological diagnosis was “compatible inflammatory changes as OLP”. Immunohistochemical examination was not performed for this biopsy sample. The lesion was monitored, and a steroid cream was prescribed at another hospital.

Two years after the first biopsy, an enlarged lesion with erythematous and induration was observed (Figure 1b). Thus, re-biopsy and consecutive total resection was performed, and a final diagnosis of “well-differentiated OSCC” was made. Based on the TNM classification of the Union for International Cancer Control [10], the histopathological stage was pT2N0M0. Six months postoperatively, symptoms of recurrence were not observed.

Written informed consent was obtained from the patient for the publication of the case report and the use of clinical photographs. This report was conducted in compliance with the Declaration of Helsinki. Based on the Japanese “Ethical Guidelines for Medical and Health Research Involving Human Subjects”, case reports are not recognized as research. Therefore, Kobe University Hospital does not require ethical approval for reporting individual cases.

## 3. Pathological Findings

Morphologically, the first biopsy showed an acanthosis with dense band-like lymphocytic infiltration in the subepithelial area, but other specific microscopic features of OLP were unclear. Cellular abnormalities were not noticeable (Figure 1c). After 2 years, the resected specimens revealed a definite 2.2-mm deep well-differentiated invasive cancer. A lymphocytic band was observed at the interface of the cancer nests (Figure 1d).

**Figure 2:**
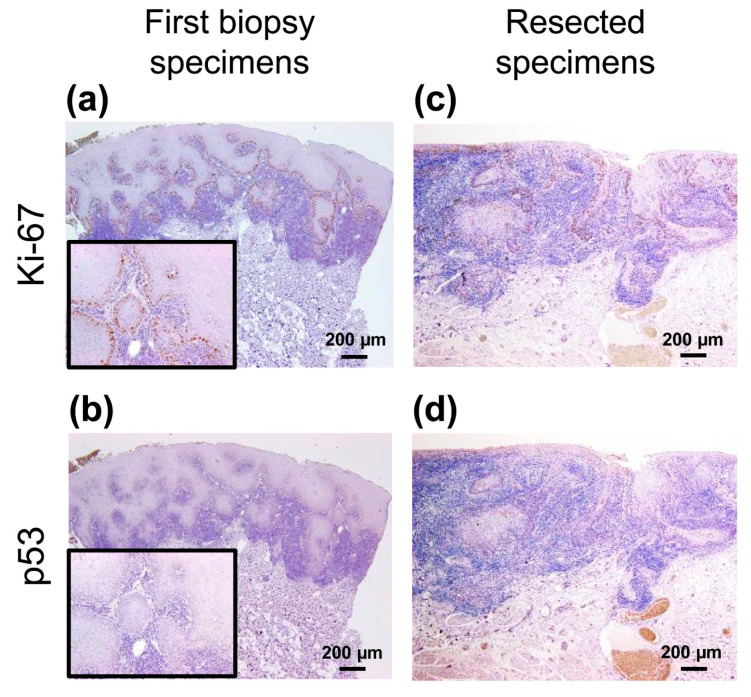
Immunohistochemical analyses of the first biopsy and resected specimens. (**a**,**c**) Ki-67^+^ cells were mainly observed in the basal cell layer in both specimens. (**b**,**d**) Few p53^+^ cells were confirmed in both the specimens. Scale bars in (**a**)–(**d**) = 200 µm. Insets in (**a**) and (**b**) show high power view of each images.

To verify whether this lesion had malignant characteristics at the first biopsy, an immunohistochemical panel was performed with Ki-67 (1:100, #MIB-1, DakoCytomation, Glostrup, Denmark), p53 (1:30, #DO-7, DAKO), and CD163 (1:100, #10D6, Novocastla, Newcastle upon Tyne, UK) retrospectively. In the first biopsy specimens, mild expression of Ki-67^+^ cells was observed in the basal or second basal layer (Figure 2a). Few p53^+^ cells were detected in the specimen (Figure 2b). Conversely, this lesion had the following immunohistochemical finding in resected specimens: Ki-67 was strongly expressed and distributed in several layers from the basal layer (Figure 2c). The p53 expression did not differ significantly from the first biopsy specimen (Figure 2d).

**Figure 3:**
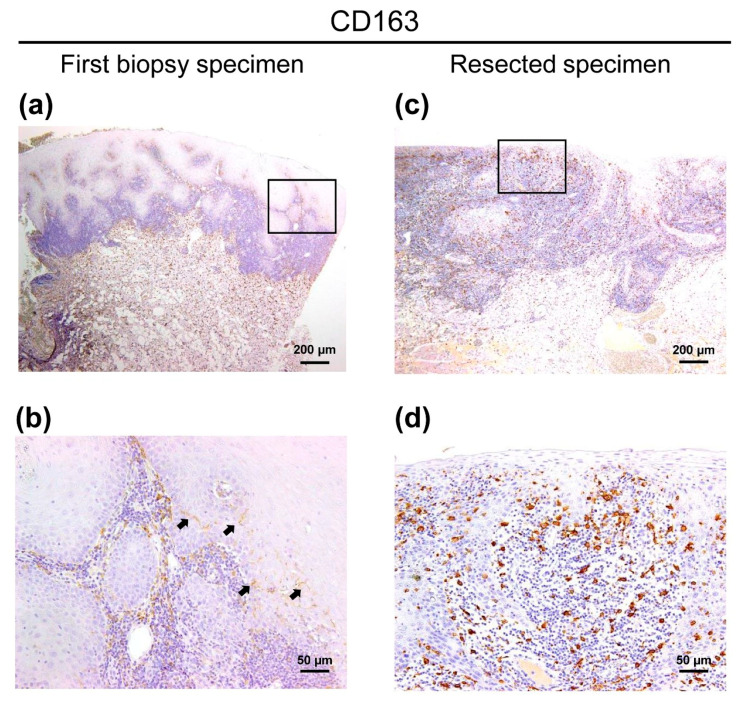
CD163 immunohistochemical images of the patient with OLC (**a**) CD163^+^ cells were distributed to not only the stromal area but also the intraepithelial area in first biopsy specimen. (**b**) High magnification view of an image of CD163-specific first biopsy specimens (square a). The infiltration of CD163^+^ cells was localized. Arrows indicate the intraepithelial CD163^+^ macrophages. (**c**) In the resected specimens, several CD163^+^ cells were diffusely infiltrated into the tumor tissue, and high expression intensity in the resected specimens was observed. (**d**) High magnification view of an image of CD163-specific resected specimens (square c). Scale bars in (**a**) and (**c**) = 200 µm. Scale bars in (**b**) and (**d**) = 50 µm.

As the evidence of malignancy in the first biopsy specimen remained unclear, CD163 expression was subsequently investigated using the same samples mentioned above. Interestingly, we observed localized infiltration of CD163^+^ cells into the epithelium in first biopsy specimens (Figure 3a,b). Additionally, the number of intraepithelial CD163^+^ cells in the resected specimens was obviously higher than in the first biopsy, and the expression intensity of the resected specimens was also stronger than that of the first biopsy (Figure 3c,d).

## 4. Discussion

We would like to stress two important points shown in this study: First, achieving an accurate evaluation of malignant potency in OLCs is difficult, not only with macroscopic but also with microscopic findings. Second, the intraepithelial CD163 was observed not only in the resected specimens but also the initial biopsy.

The unilateral lichenoid condition was initially diagnosed as OLP clinically. Simultaneously, the histological diagnosis of compatible change as OLP was established by the first biopsy. OLL tends to display a unilateral distribution as compared with OLP [1]. Most concerns on the clinical aspects related to the risk of malignant development in patients with OLP are associated with atrophic-erosive lesions in the tongue [11]. However, in this case, erosive changes of the lesion were not displayed. Therefore, we should be aware of the limitation to evaluate the malignant potency of OLC based on its clinical appearance only.

Additionally, this lesion had mild atypia recognized as a reactive change based on morphological evaluation using a hematoxylin and eosin (HE)-stained specimen. Rock et al. proposed that dysplasia should not be ignored in lichenoid mucositis [12]. Moreover, OSCC has a well-differentiation tendency, and the cell atypia is often mild. It has also been reported that the evaluation of oral epithelial dysplasia is subjective [13]. These previous reports indicate that malignant potency of the unilateral OLC should be more carefully evaluated by both clinicians and pathologists.

We conducted a retrospective immunohistochemical analysis. In this case, a definite immunoreactivity of Ki-67 and p53 as indicators of malignancy was not observed. Although it may be difficult to correctly diagnose OLC based on the microscopic findings of conventional markers, we must recognize that only limited conclusions can be drawn from this single case report.

We previously proposed that tongue leukoplakia biopsy, with an intraepithelial lesion in which intraepithelial CD163^+^ cells are observed, may suggest that invasive cancer is present in the neighborhood of the specimen [9]. We herein sought to determine whether CD163^+^ macrophages provide a hint for the evaluation of malignant potency of OLC. In this case, intraepithelial CD163^+^ macrophages were detected in both specimens. Remarkably, the intensity of CD163 in the resected specimen was also higher than that of the biopsy specimen. We speculate that the biological behavior of CD163^+^ macrophages differs greatly depending on localization and immunoreactive intensity in oral carcinogenesis.

From oncological viewpoints, activated macrophages are classified as antitumor M1 and pro-tumor M2 types [14,15,16]. In oral malignant tissue, M1 macrophages express CD11c or CD80 [17,18,19], whereas CD163 is the most suitable marker for M2 macrophages [20,21,22]. Weber et al. proposed that high epithelial versus subepithelial expression ratio of both CD163 and CD11c^+^ macrophages was correlated with the malignant transformation of oral leukoplakia [17]. The immunohistochemical findings of CD163 in their report support our results. Moreover, they demonstrated that the intraepithelial CD163/CD11c^+^ ratio is significantly higher in oral leukoplakia with malignant transformation than in oral leukoplakia without transformation [17]. Although we also assessed the presence of M1 macrophages in the present case, conclusive findings could not be obtained (data not shown). Unlike that for M2 macrophages, no suitable immunohistochemical markers have been identified for detecting M1 macrophages [15]. Therefore, comparisons with other OLC samples using plural M1 markers are required to test whether the malignant potential of OLC could be detected using the M2/M1 ratio.

In this case, whether this patient had invasive cancers initially, or the malignant transformation occurred after the first biopsy, must be carefully discussed. Previously, we demonstrated CD163^+^ macrophages were infiltrated in the stromal area, but not in the intraepithelial area of nonneoplastic lesions including OLP, oral candidiasis and aphthous stomatitis [9]. Our findings, overall, indicate that OLC in this study had a high malignant potency, and the small number of intraepithelial CD163^+^ macrophages observed in the first biopsy specimen should not be overlooked.

To the best of our knowledge, no previous reports have evaluated CD163^+^ macrophage counts in OLC with malignant potency using both biopsy and resected specimens. However, caution must be exercised when interpreting the results of a single case report. A previous meta-analysis of 20095 patient data reported that OLP affects 1–4% of the population worldwide, and only 1.1% of them develop OSCC. Moreover, it may not be possible to completely distinguish OLP from other lichenoid lesions in included studies [23]. Therefore, the investigation of such a small number of cases of malignant transformation of OLP is difficult. In fact, a search for the other cases of OLC with malignant potential using the Kobe University Hospital Pathology System extracted no additional cases. To clarify the involvement of intraepithelial CD163^+^ macrophages in the malignant characteristics of OLC, prospective studies using both OLC samples and healthy control samples are needed.

In conclusion, this is the first report to demonstrate that intraepithelial CD163^+^ macrophages are a noteworthy indicator to evaluate the malignant potency in patients with OLC.
